# Engineering of Nanobodies Recognizing the Human Chemokine Receptor CCR7

**DOI:** 10.3390/ijms20102597

**Published:** 2019-05-27

**Authors:** Barbara D. Jakobs, Lisa Spannagel, Vladimir Purvanov, Edith Uetz-von Allmen, Christoph Matti, Daniel F. Legler

**Affiliations:** 1 Biotechnology Institute Thurgau (BITg) at the University of Konstanz, CH-8280 Kreuzlingen, Switzerland; barbara.jakobs@bitg.ch (B.D.J.); lisa.spannagel@bitg.ch (L.S.); vladimir.purvanov@bitg.ch (V.P.); edith.uetz@bitg.ch (E.U.-v.A.); christoph.matti@bitg.ch (C.M.); 2 Graduate School for Cellular and Biomedical Sciences, University of Bern, CH-3012 Bern, Switzerland; 3 Faculty of Biology, University of Konstanz, D-78464 Konstanz, Germany

**Keywords:** chemokine receptor, CCR7, chemokines, CCL19, nanobodies, β_2_-adrenergic receptor, bimolecular fluorescence complementation, split-luciferase complementation

## Abstract

The chemokine receptor CCR7 plays a pivotal role in health and disease. In particular, CCR7 controls homing of antigen-bearing dendritic cells and T cells to lymph nodes, where adaptive immune responses are initiated. However, CCR7 also guides T cells to inflamed synovium and thereby contributes to rheumatoid arthritis and promotes cancer cell migration and metastasis formation. Nanobodies have recently emerged as versatile tools to study G-protein-coupled receptor functions and are being tested in diagnostics and therapeutics. In this study, we designed a strategy to engineer novel nanobodies recognizing human CCR7. We generated a nanobody library based on a solved crystal structure of the nanobody Nb80 recognizing the β_2_-adrenergic receptor (β_2_AR) and by specifically randomizing two segments within complementarity determining region 1 (CDR1) and CDR3 of Nb80 known to interact with β_2_AR. We fused the nanobody library to one half of split-YFP in order to identify individual nanobody clones interacting with CCR7 fused to the other half of split-YFP using bimolecular fluorescence complementation. We present three novel nanobodies, termed Nb1, Nb5, and Nb38, that recognize human CCR7 without interfering with G-protein-coupling and downstream signaling. Moreover, we were able to follow CCR7 trafficking upon CCL19 triggering using Nb1, Nb5, and Nb38.

## 1. Introduction

The system of chemokine receptors and their ligands, the chemokines, is crucial for guiding cell migration in development, health, and disease. Chemokines are small, secreted chemotactic cytokines that play a major role in tightly coordinating the migration and positioning of immune cells, thereby essentially contributing to both development of the immune system and regulation of innate and adaptive immune responses [1,2,3]. However, chemokines also orchestrate cancer cell dissemination and metastasis formation [4]. As a consequence, chemokines and their receptors have emerged as therapeutic targets, particularly in immune and inflammatory disorders as well as in cancer [5].

The chemokine receptor CCR7, together with its ligands, CCL19 and CCL21, orchestrates the migration of antigen-loaded dendritic cells (DCs) and lymphocytes to lymphoid organs to launch specific immune responses against invading pathogens [6,7]. CCL21 is constantly produced by lymphatic endothelial cells in peripheral tissues forming immobilized chemokine gradients from the interstitium towards lymphatic vessels [8]. Upon pathogen encountering in peripheral tissues, DCs induce the expression of CCR7 facilitating their migration along local chemokine gradients and subsequent homing to draining lymph nodes. Naïve T cells constantly express CCR7 and recirculate between the bloodstream, lymphoid organs and lymphatics in search for cognate antigens. They enter lymph nodes through high-endothelial venules presenting CCR7 ligands. In lymph nodes, CCL19 and CCL21 are constitutively expressed by fibroblastic reticular cells in the T cell zone, where antigen-bearing DCs and circulating T cells encounter each other in a CCR7-dependent manner [6]. Notably, misguidance of leukocytes in mice lacking either CCR7 or its ligands was found to facilitate multi-organ autoimmunity [9]. Moreover, CCL21 is also produced by endothelial cells of rheumatoid synovial tissue where CCR7-expressing effector T cells are retained in the inflamed synovium to contribute to disease progression [10]. Finally, CCR7 is also expressed by numerous cancer cell types and promotes cancer cell migration, dissemination, and metastasis formation in lymphoid organs [11].

Chemokine receptors belong to the class A of heptahelical G-protein-coupled receptors (GPCRs) [12]. Chemokine receptor signaling in general is initiated by binding of its ligands to the receptor, thereby inducing the GDP/GTP exchange of coupled heterotrimeric G_i_-proteins. This leads to the dissociation of the βγ-subunits from the Gα_i_-subunit, both of which are able to activate further downstream signaling cascades [12]. In particular, Gα-GTP is responsible for reassembly and regeneration of the inactive heterotrimeric G-protein, whereas free βγ-subunits activate phospholipase C and phosphoinositide-3-kinase. Activation of these effector enzymes is followed by activation of both second messengers, including calcium ions from intracellular stores, and different kinases, such as the extracellular signaling regulated kinases Erk-1/2 [13,14,15]. For CCR7, receptor oligomerization was shown to facilitate the interaction with Src kinase, which becomes activated upon chemokine triggering, resulting in tyrosine phosphorylation of the conserved DRY motif of the receptor [13,14,16]. This tyrosine phosphorylation by Src kinase establishes a docking site for the tyrosine phosphatase SHP2 [16] and the tyrosine kinase ZAP70 [17] independent of Gα_i_-coupling. Responsiveness of CCR7 is regulated by the recruitment of GPCR kinases (GRKs) that phosphorylate the C-terminus of the receptor at multiple serine and threonine residues resulting in β-arrestin binding and receptor internalization [13]. Noteworthy, only CCL19, but not CCL21, promotes robust β-arrestin recruitment and thus receptor internalization [14].

Recent X-ray structures of a number of GPCRs [18] together with structural information on the chemokine receptor CXCR4 [19] and CCR5 [20] in complex with a ligand provided new insights in how chemokine receptors may transmit signals across the plasma membrane. Ligand binding to the extracellular domains of the receptor in cooperation with the local membrane microenvironment drives conformational changes along transmembrane helices to the intracellular domains of the GPCR that instigate downstream signaling [18]. To gain insights into the structure-function relationship of the prototype class A GPCR, the β_2_-adrenergic receptor (β_2_AR), a camelid antibody fragment referred to as nanobody (Nb), was generated [21]. This was achieved by immunizing Llama with purified agonist-bound β_2_AR and revealed the nanobody Nb80. Nb80 was found to interact with activated β_2_AR and to exhibit G-protein-like behavior by mimicking the energetic coupling of agonist and Gα_s_-binding and enabled to obtain an active-state crystal structure of β_2_AR [21]. Subsequently, Nb80 fused to GFP was used as a conformational biosensor to monitor activation of β_2_AR revealing presence of active β_2_AR at the plasma membrane as well as within membranes of early endosomes [22]. As Nbs can be easily produced as recombinant minimal-sized, single domain protein harboring the full antigen binding capacity, in combination with a compact prolate shape and ideal biochemical characteristics, such as solubility, thermal and conformational stability, Nbs represent a promising tool not only to study GPCR structures and functions but also in cancer diagnosis and therapy [23,24,25].

Here, we established a new strategy to identify Nbs recognizing human CCR7 by generating a randomized Nb library based on the sequence and structure of Nb80 and by subsequent screening for suitable Nb clones interacting with CCR7 using bimolecular fluorescence complementation (BiFC).

## 2. Results

### 2.1. Engineering of Nbs Recognizing Human CCR7

**Figure 1:**
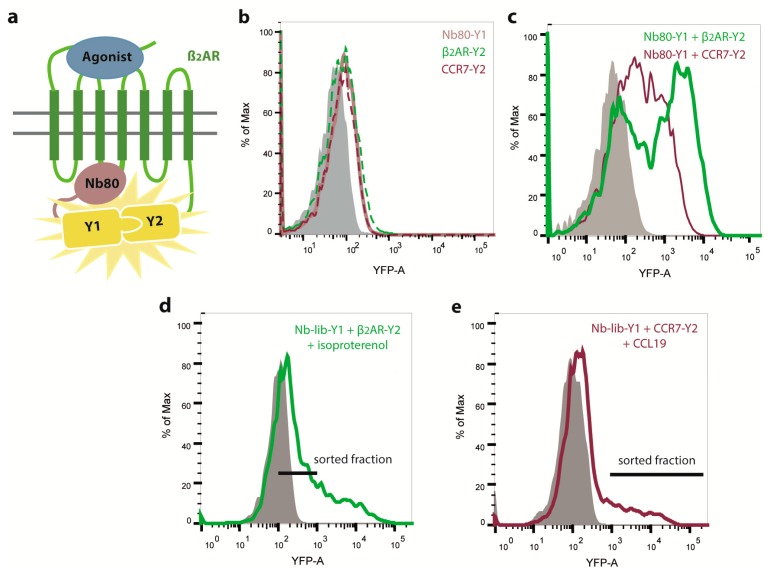
Engineering of nanobodies (Nbs) recognizing CCR7 by bimolecular fluorescence complementation (BiFC). Structure and sequence of the conformation-specific Nb80, which recognizes an isoproterenol-activated conformation of the β2-adrenergic receptor (β_2_AR), was used to specifically randomize complementarity determining region (CDR)1 and CDR3 to generate a new Nb library. The newly generated Nb library was fused to the N-terminal part of split-YFP (YFP1, Y1) in order to identify Nbs that recognize CCR7 fused to split-YFP2 (Y2) by BiFC. (**a**) Schematic illustration of BiFC between Nb80-YFP1 and β_2_AR-YFP2. Nb80 recognizes and binds to agonist activated β_2_AR. Thereby, the two split-YFP fragments will reconstitute to form native YFP. (**b**) Flow cytometric analysis of HEK293 cells transiently expressing either Nb80-YFP1 (dotted brown line), β_2_AR-YFP2 (dotted green line), or CCR7-YFP2 (dotted red line) alone. Untransfected, control cells are shown in grey. (**c**) Flow cytometric analysis showing BiFC in HEK293 cells transiently co-expressing Nb80-YFP1 and β_2_AR-YFP2 (green line) as proof of concept or Nb80-YFP1 and CCR7-YFP2 (red line) as control. Before measuring YFP fluorescence, cells were stimulated with isoproterenol (10 µM) or CCL19 (0.5 µg/mL), respectively. (**d**) Negative screening of Nb library to remove β_2_AR-recognizing Nbs. HEK293 cells were transiently transfected with the newly generated Nb library fused to split-YFP1 (Nb-lib-Y1) and β_2_AR fused to split-YFP2. After isoproterenol stimulation (10 µM), BiFC-negative cells were FACS sorted to enrich the Nb library for Nbs that do not interact with β_2_AR anymore. Sorted cell fraction is indicated by the black line. Afterwards, plasmids coding for the Nb library were isolated. (**e**) BiFC of remaining Nb library-YFP1 and CCR7-YFP2. The Nb library-YFP1 was transiently expressed in HEK293 cells stably expressing CCR7-YFP2 and cells were stimulated with CCL19 (0.5 µg/mL). BiFC-positive cells, indicated by the black line, were FACS sorted.

Nbs recently emerged as attractive and versatile tools for research purposes [21,23,24,26] and are being tested in diagnostics and therapeutics [27]. Nbs possess many advantages over conventional antibodies, including their small size, thermal stability and unique three-dimensional structure, which allows binding to cavities or clefts on the surface of proteins that are mostly inaccessible to conventional antibodies. Most prominently, nanobody Nb80 is considered as conformation-specific Nb recognizing active β_2_AR [21]. Crystallography revealed that Nb80 especially binds with the third complementarity determining region (CDR3) to the cytoplasmic end of β_2_AR and protrudes into the core of the receptor [21]. More precisely, an eight amino acid long sequence of CDR3 penetrates into a hydrophobic pocket established by amino acids of the receptor’s transmembrane helices 3, 5, 6, and 7. In addition, a four amino acid sequence of the Nb80′s CDR1 stabilizes the interaction with regions of helices 5 and 6 of the GPCR [21]. The interaction of Nb80 with β_2_AR stabilizes a conformational state that highly resembles the active state of isoproterenol-stimulated receptor in complex with the G-protein [21,26]. To avoid the need of immunizing Llama and thus the requirement of large amounts of purified, reconstituted and activated CCR7, we intended to engineer Nbs recognizing CCR7 by taking advantage of the conserved structural architecture of GPCRs and the detailed structural information, which is available for Nb80. More precisely, we applied synthetic randomization to CDR1 and CDR3 of Nb80 with the aim to lose the affinity of new Nbs for β_2_AR while gaining specificity to CCR7, combined with the powerful and highly sensitive BiFC approach [16,28]. To this end, we cloned the newly generated Nb cDNA library in frame to the coding sequence of the N-terminal part of split-YFP1. By co-expressing the Nb library fused to YFP1 together with CCR7 fused to split-YFP2, we anticipated to identify Nbs that interact with human CCR7 by BiFC. Furthermore, we stimulated CCR7 with CCL19 hoping to also identify Nbs recognizing an active conformation of the receptor. As proof of principle for detecting Nb-GPCR interaction by BiFC, we first co-expressed Nb80-YFP1 together with β_2_AR-YFP2 in HEK293 cells and stimulated the cells with isoproterenol, a ligand of β_2_AR (Figure 1a). Notably, no BiFC was detected in HEK293 cells transfected with only Nb80-YFP1, β_2_AR-YFP2, or CCR7-YFP2 (Figure 1b). However, we observed BiFC between Nb80-YFP1 and β_2_AR-YFP2 (Figure 1c). By co-expressing Nb80-YFP1 and CCR7-YFP2, we noted some interaction, but the YFP fluorescence intensity was much weaker compared to the one derived from the Nb80-YFP1 and β_2_AR-YFP2 BiFC (Figure 1c).

**Figure 2:**
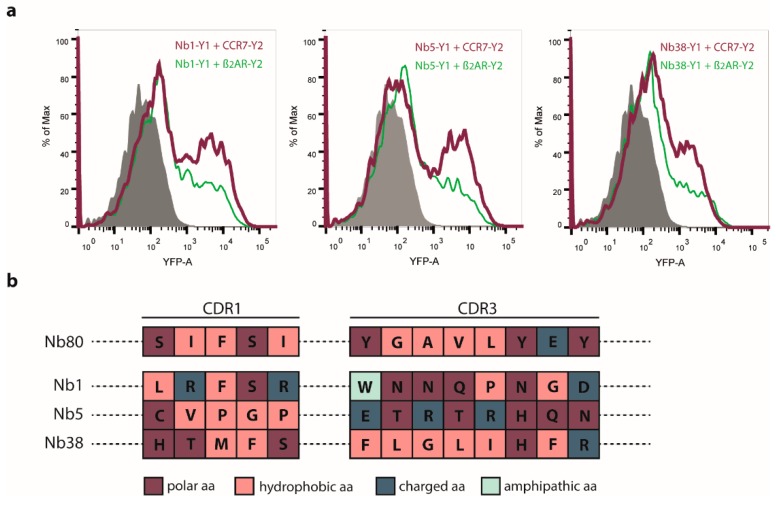
Flow cytometric analysis (**a**) and sequence analysis (**b**) of individual Nb clones previously selected by BiFC screening. (**a**) Plasmids coding for Nb-YFP1 (Y1) were isolated and individual clones were co-transfected again with CCR7-YFP2 (Y2) to screen for single Nb clones that recognize CCR7. Here, the three most promising Nb clones are represented: Nb1, Nb5, and Nb38. BiFC between individual Nb clones and CCR7 is indicated in red. Additionally, BiFC of individual Nb-YFP1 clones and β_2_AR-YFP2 was analyzed and is depicted in green. (**b**) Nb1, Nb5, and Nb38 were sequenced. Protein sequences of CDR1 and CDR3 of each Nb are illustrated in comparison to Nb80. Different colors were used to highlight characteristic properties of respective amino acids (aa).

This prompted us to first implement an initial negative screening, in which we transfected cells with our Nb library-YFP1 together with β_2_AR-YFP2, sorted for BiFC-negative cells (Figure 1d) and isolated plasmids coding for the Nb library. The remaining Nb library fused to YFP1 was subsequently transfected into HEK293 cells stably expressing CCR7-YFP2, stimulated with CCL19 and BiFC-positive cells were sorted (Figure 1e). Plasmids coding for Nb-YFP1 were isolated and individual clones were co-transfected again with CCR7-YFP2. The three most promising Nb clones, referred to as Nb1, Nb5, and Nb38 (Figure 2a), were sequenced and further analyzed. As observed for Nb80 and CCR7, the CCR7-recognizing Nbs retain some basal interaction with β_2_AR (Figure 2a). This might be explained by the fact that GPCRs flicker between different conformational states and that protein-protein interactions within the BiFC system are relatively long-lived.

Comparing the CDR1 and CDR3 regions of the three selected CCR7-recognizing Nb clones revealed that the two binding domains of Nb38 mainly consist of hydrophobic amino acids similar to the CDR1 and CDR3 region of Nb80 (Figure 2b). Notably, the binding domains of Nb1 and Nb5 contain more polar amino acids, and in the case of Nb5, include charged residues.

### 2.2. Nb1, Nb5, and Nb38 Preferentially Recognize CCR7 While Nb80 Preferentially Interacts with β_2_AR

**Figure 3:**
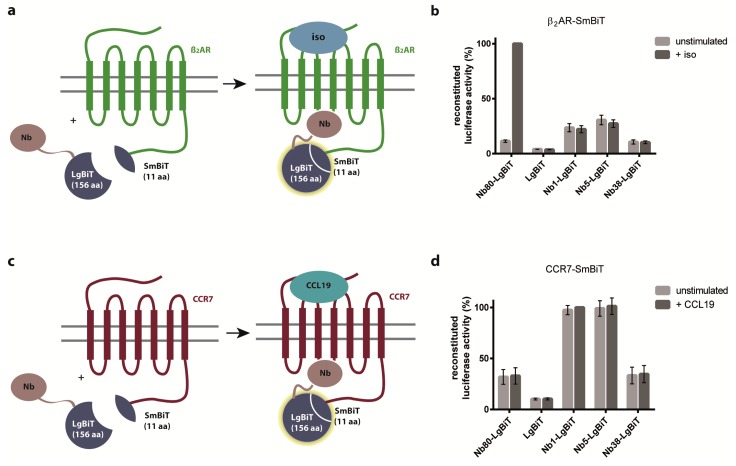
Nb80 preferentially interacts with active β_2_AR, whereas Nb1, Nb5, and Nb38 preferentially recognize CCR7 independent of its activation state as assessed by split-luciferase complementation. (**a,c**) Schematic representation of the split-luciferase complementation assay. Nb-GPCR interactions are determined by reconstitution of Small BiT (SmBiT) and Large BiT (LgBiT) to functional NanoLuc (NLuc) luciferase before and after agonist stimulation and subsequent measurements of luminescence signals generated by the reconstituted luciferase. (**b,d**) HEK293 cells transiently co-expressing β_2_AR (**b**) or CCR7 (**d**) fused to SmBiT of NLuc and individual Nb clones fused to LgBiT of NLuc were incubated with coelenterazine H (5µM), the luciferase’s substrate, and after 10 min, stimulated with isoproterenol (iso) (10 µM) (**b**) or CCL19 (1.5 µg/mL) (**d**). As control, we transiently co-expressed LgBiT without Nb together with either GPCR-SmBiT. Reconstituted luciferase activity between Nb80 and β_2_AR (**b**) and Nb1 and CCR7 (**d**), respectively, was set to 100%. Results represent each the mean values of three independent experiments including the standard error of the mean (SEM).

To circumvent the limitations of the BiFC system, we next established a method that allows determining more transient and dynamic protein-protein interactions. To this end, we applied a split-luciferase complementation assay, in which we co-expressed either β_2_AR fused to Small BiT (SmBiT) (Figure 3a) or CCR7-SmBiT (Figure 3c) together with Nb fused to Large BiT (LgBiT) of the NanoLuc (NLuc) luciferase in HEK293 cells. The advantage of this system is that the luciferase falls apart into the split parts if the fused proteins of interest no longer interact with each other. Exploiting this split-luciferase complementation assay revealed that Nb80 predominantly interacted with activated β_2_AR (Figure 3b), whereas the newly engineered Nb1, Nb5, and Nb38 predominantly recognized CCR7 independent of its activation state (Figure 3d).

### 2.3. Nb1, Nb5, and Nb38 Barely Interfere with G-Protein-Coupling to CCR7

**Figure 4:**
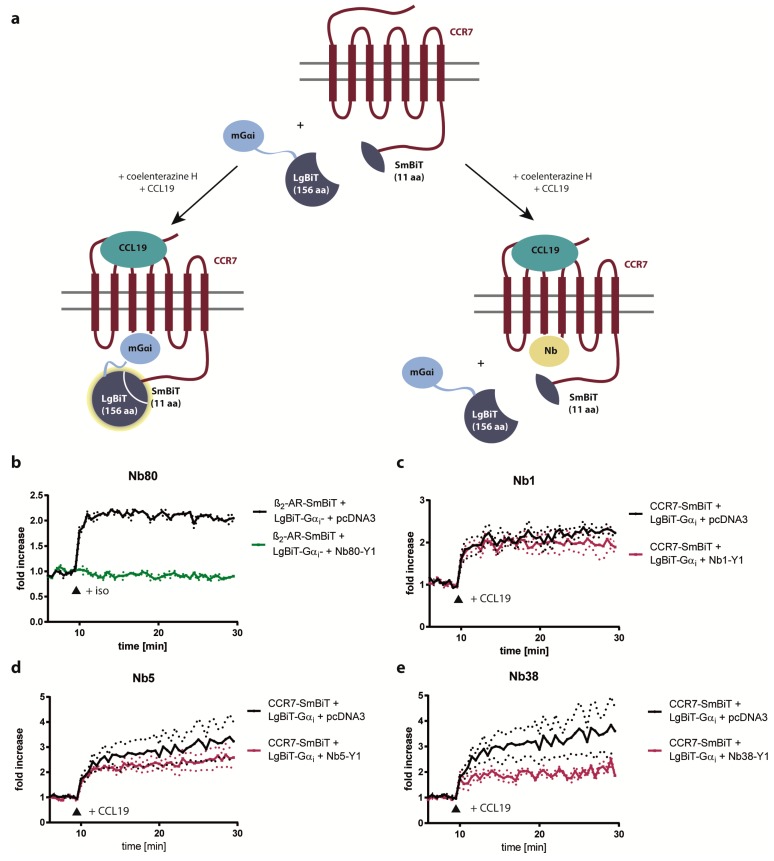
CCL19-triggered G-protein coupling to CCR7 is barely impaired by Nb1, Nb5, or Nb38. (**a**) Schematic illustration of the G-protein competition assay based on split-luciferase complementation. (**b**–**e**) HEK293 cells transiently expressing β_2_AR-SmBiT (**b**) or CCR7-SmBiT (**c**–**e**) together with LgBiT-mGα_i_ and Nb-YFP1 (Nb-Y1) constructs were incubated with coelenterazine H (5 µM) and subsequently stimulated (indicated by the arrow head) either with 10 µM isoproterenol (**b**) or 1.5 µg/mL CCL19 (**c**–**e**), respectively. As control, HEK293 cells were transiently co-transfected with GPCR-SmBiT, LgBiT-mGα_i_ and pcDNA3 (indicated in black). In this case, increase in luminescence indicated functional activity of reconstituted split NLuc luciferase upon recruitment and interaction of mGα_i_ with the GPCR. Replacing empty vector (pcDNA3) with Nb80-YFP1 (**b**) caused complete blockage in luciferase activity (indicated in green). Expressing Nb1-YFP1 (**c**), Nb5-YFP1 (**d**) or Nb38-YFP1 (**e**) barely interfered with mGα_i_-coupling to CCR7 (indicated in red). Results represent the fold increase in luminescence over the baseline, which is set to 1, upon agonist-stimulation. Mean values and SEM of three independent experiments are shown.

As Nb80 binding to agonist-activated β_2_AR is known to inhibit G-protein activation and consequently interferes with downstream signaling [29], we further examined the possibility of CCR7-interacting Nbs to interfere with CCR7-driven G-protein-coupling. To achieve this, we conducted a novel G-protein competition assay based on split-luciferase complementation (Figure 4a). As proof of concept, we first tested the inhibitory capacity of Nb80 on G-protein-coupling to β_2_AR upon isoproterenol stimulation. In fact, Nb80 completely blocked G-protein interaction with ligand-stimulated β_2_AR (Figure 4b). In contrast, Nb1, Nb5, and Nb38 barely interfered with CCL19-driven G-protein-coupling to CCR7 (Figure 4c–e).

### 2.4. Nb1, Nb5, or Nb38 do not Impair CCR7-Driven Calcium Mobilization and Receptor Endocytosis

**Figure 5:**
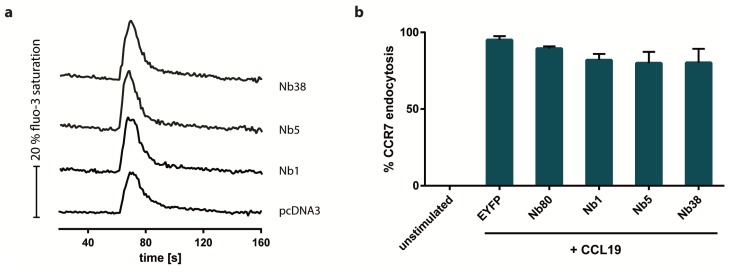
CCL19-mediated calcium mobilization and CCR7 endocytosis are not affected by Nb1, Nb5, or Nb38. (**a**) Influence of intracellular expression of the CCR7-recognizing Nbs, Nb1, Nb5, and Nb38, on CCL19-mediated changes in intracellular calcium concentrations was measured in H1299 cells stably expressing CCR7-HA. Cells were stimulated with 0.5 µg/mL of CCL19. A representative experiment out of three is shown. (**b**) Endocytosis of CCR7 triggered by 1 µg/mL CCL19 was analyzed in HEK293-CCR7-HA cells transiently expressing CCR7-recognizing Nbs. Co-expressing EYFP or Nb80 instead of CCR7-recognizing Nbs served as controls. Mean values and SEM out of three independent experiments are presented.

To further characterize the newly engineered CCR7-recognizing Nbs, we next determined whether the Nbs interfere with chemokine-driven calcium mobilization and receptor internalization. Our results revealed that none of the CCR7-recognizing Nbs interfered with either CCL19-mediated mobilization of calcium ions from intracellular stores (Figure 5a) or CCL19-mediated CCR7 endocytosis (Figure 5b). These data provide clear evidence that CCR7 signaling is not compromised in the presence of the newly engineered CCR7-recognizing Nbs.

### 2.5. Monitoring CCL19-induced CCR7 trafficking by Nb1, Nb5, and Nb38

**Figure 6:**
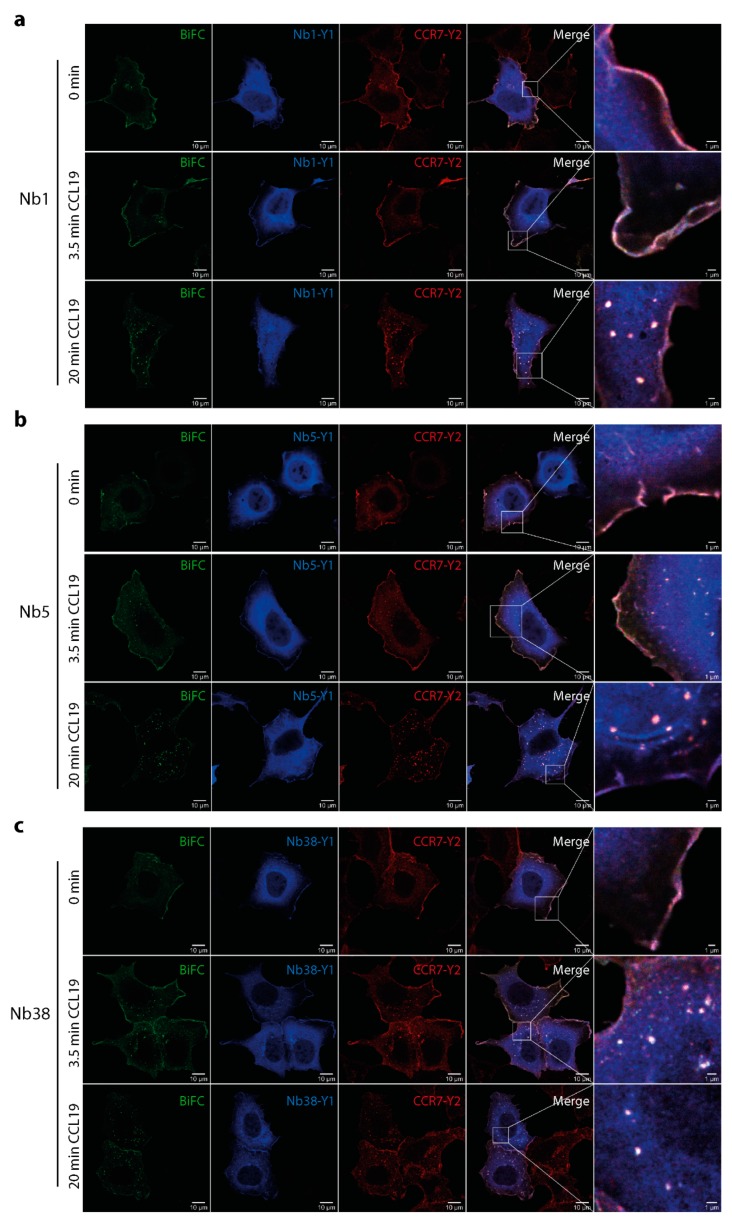
Monitoring CCR7 trafficking triggered by CCL19 using Nb1, Nb5, and Nb38. Interaction between CCR7-YFP2 (Y2) and individual Nbs fused to YFP1 was assessed by BiFC and confocal microscopy in HEK293 transfected cells that were unstimulated or stimulated for indicated time points with 0.5 µg/mL of CCL19. Nb1-YFP1 (Y1) (**a**), Nb5-YFP1 (Y1) (**b**), and Nb38-YFP1 (Y1) (**c**). In addition, Nb-Y1 and CCR7-Y2 were immunostained using anti-YFP1 and anti-YFP2 specific antibodies. Scale bar: 10 µm, zoomed image: 1 µm.

Finally, we assessed the newly engineered Nbs for their capacity to detect CCR7 at the plasma membrane and at endocytic vesicles upon chemokine stimulation by confocal microscopy. In line with our BiFC assays shown in Figure 1, we observed that Nb1 (Figure 6a), Nb5 (Figure 6b) and Nb38 (Figure 6c) interacted with CCR7 by BiFC. Notably, in the absence of ligands, BiFC between the three Nb-YFP1 clones and CCR7-YFP2 was primarily observed at the plasma membrane. Upon CCL19 stimulation, BiFC was also found at membrane ruffles and in vesicular structures. Latter was most pronounced after prolonged stimulation with chemokine.

Taken together, in the present study we designed a novel strategy to successfully engineer Nbs interacting with the chemokine receptor CCR7. We found that a selection of newly engineered Nbs recognize human CCR7 independent of its activation state and without interfering with G-protein-coupling, chemokine-mediated calcium mobilization or receptor internalization, but allow following CCR7 trafficking upon chemokine engagement in space and time.

## 3. Discussion

The chemokine receptor CCR7 plays a crucial role in guiding migration of immune cells, particularly of DCs and T cells, into secondary lymphoid organs to launch adaptive immune responses [6,7,14]. However, CCR7-mediated cell migration also contributes to inflammatory diseases, such as rheumatoid arthritis [10], or facilitates metastasis of cancer cells [11]. Moreover, misguidance of immune cells due to impaired CCR7 signaling may lead to autoimmune diseases [9]. Despite the well appreciated central role of CCR7-driven cell migration in health and disease, molecular mechanisms how CCR7 signaling controls cell migration are far from being understood. Hence, new molecular insights into how CCR7 signaling guides cell migration are highly desired. To gain such molecular insights, new tools are required to assess and monitor CCR7 signaling.

In this study, we designed a strategy to develop Nbs recognizing CCR7. Using this strategy, we successfully engineered Nbs recognizing human CCR7 by synthetic randomization of the binding domains CDR1 and CDR3 of the β_2_AR conformation-specific Nb80. In order to identify single Nb clones that interact with CCR7 out of a Nb library of more than a million clones, we exploited a high-throughput BiFC approach combined with single cell sorting. We subsequently selected individual CCR7-recognizing Nbs based on the ability to interact with CCR7 over β_2_AR by using a split-luciferase.

Nb80 was isolated and identified upon immunization of Llama with purified, agonist-bound β_2_AR that was reconstituted at high density into phospholipid vesicles [21]. To our knowledge, no one has achieved to purify CCR7 in sufficient amounts to immunize Llamas to generate CCR7-specific nanobodies, not spoken of the need to complex the purified receptor with a ligand for immunization to potentially get nanobodies reacting with active receptors. Notably, Nb80 stabilizes the active conformation of β_2_AR, which is ideal for crystallization studies. Due to its high affinity, Nb80, however, interferes with G-protein-coupling of β_2_AR and consequently dampened agonist-driven cAMP production and β-arrestin recruitment [29] (Figure 4b). In contrast, our CCR7-recognizing Nbs do not discriminate between inactive and agonist-stimulated states of the receptor and barely interfere with chemokine-mediated G-protein-coupling (Figure 4) or downstream signaling (Figure 5). Hence, it is unlikely that our Nbs recognizing CCR7 stabilize the receptor in a particular conformation. Moreover, as no structural information is available for CCR7, it remains to be determined how similar G-protein-coupling to CCR7 and β_2_AR is. However, it is reasonable that differences between Nb80 binding to β_2_AR and Nb1/5/38 binding to CCR7 are due to alternative biochemical properties of the polypeptide chains within CDR1 and CDR3 of individual Nb clones and the GPCR. Notably, Nb1 includes a glycine residue in CDR3, which confers high flexibility, whereas Nb5 possesses more charged amino acids in CDR3. Interestingly, the presence of proline in Nb1 gives a hint that protrusion of the long CDR3 loop into the receptor cavity might be impeded. This is consistent with the observation that Nb1 did not affect G-protein-coupling at all whereas Nb5 and Nb38 slightly reduced G-protein-coupling to CCR7. We used, in this assay, mini-Gα_i_ (mGα_i_)-proteins, which functionally mimic the nucleotide-free G-protein bound to GPCR, as surrogate for heterotrimeric G-proteins since these mG-proteins are reported as excellent sensors for activation of GPCRs [30]. Since CCR7 predominantly couples to Gα_i_, we used the same mG-protein also for the positive control, the β_2_AR, even though it is well established that β_2_AR preferentially couples to Gα_s_ but secondarily also couples to Gα_i_ just with lower potency [30]. Despite this inhibitory role in β_2_AR signaling, Nb80 fused to GFP was successfully used as biosensor to identify active β_2_AR at both the plasma membrane and subsequently at endosomes upon agonist triggering [22]. Similarly, our newly generated Nbs recognized CCR7 at the plasma membrane and, upon CCL19 triggering, also at endocytic vesicles (Figure 6).

Finally, our newly developed strategy to engineer Nbs can be used and further developed to generate additional Nbs for other GPCRs and might foster the use of such Nbs for future diagnostic and therapeutic purposes.

## 4. Materials and Methods

### 4.1. Reagents and Antibodies

Recombinant human CCL19 and CCL21 were purchased from PeproTech (Rocky Hill, CT, USA), ionomycin and the chemical isoproterenol hydrochloride were obtained from Sigma-Aldrich (St. Louis, MO, USA), fluo-3-AM was from Molecular Probes (Eugene, OR, USA), whereas the luminescence substrate coelenterazine H (2-(4-Dehydroxy) coelenterazine) was purchased from Biosynth (Staad, Switzerland). All restriction enzymes were purchased from Thermo Fischer Scientific (Waltham, MA, USA). The following antibodies were used for immune fluorescence: anti-YFP1 (E385) (abcam, Cambridge, United Kingdom), anti-YFP2 (11814460001) (Roche, Basel, Switzerland), goat anti-rabbit IgG (H+L) cross-adsorbed secondary antibody Alexa Fluor^®^ 568 (A11011) (Invitrogen, Carlsbad, CA, USA) and goat anti-mouse IgG (H+L) cross-adsorbed secondary antibody Alexa Fluor ^®^ 647 (A21235) (Life Technologies, Carlsbad, CA, USA). For flow cytometry analysis of CCR7 endocytosis anti-human CCR7 APC-conjugated antibody (FAB197A) (R&D Systems, Minneapolis, MN, USA) and IgG2A APC-conjugated antibody, as isotype-matched control, (IC003A) (R&D Systems) were used.

### 4.2. Cell Lines and Transfection

HEK293 cells as well as H1299 cells stably expressing CCR7-HA [15,31] were cultured in DMEM containing 10% FCS (Gibco, Waltham, MA, USA) and 1% penicillin/streptomycin (Lonza, Basel, Switzerland). HEK293 cells were stably transfected with CCR7-YFP2 using TransIT-LT-1 (MirusBio, Madison, WI, USA) as specified by the manufacturer and selection with G418 (Gibco). HEK293 cells were transiently transfected with 1µg plasmid DNA and 3µl FuGENE^®^6 Transfection Reagent (Promega Corporation, Madison, WI, USA) according to the manufacturer’s instructions and analyzed 24–48 h post transfection, as specified in the corresponding assays.

### 4.3. Construction of Expression Plasmids

The plasmid pEGFP-N1-Nb80 was kindly provided by Mark von Zastrow [22]. Full length Nb80 was amplified by PCR (template: pEGFP-N1-Nb80) using specific primers (forward: 5′ ATC TCG AGC TCA AGC TTG CCG CCA CCA TGG GAC AG 3′; reverse: 5′ ATT ACA TCG ATG TGA TGG TGA TGG TGG TGT AGA G 3′) and subcloned into the previously described C-terminally tagged split-YFP1 BiFC vector [16,28] by using the restriction enzymes HindIII and ClaI. Cloning of pcDNA3-CCR7-YFP2 has been described previously [17]. Briefly, CCR7 was amplified by PCR and subcloned into the EcoRI and ClaI sites of the split-YFP2 BiFC vector [16,28]. For cloning of pcDNA3-β_2_AR-YFP2, the full length sequence of human ADRB2 (β_2_AR) was amplified by PCR using pCMV6-XL5-β_2_AR purchased from OriGene (Rockville, MD, USA) as a template and a specific primer pair (forward: 5′ CTA CGA ATT CAG CCG CTG AAT GAG GCT T 3′; reverse: 5′ CGG CAT CGA TTA GCA GTG AGT CAT TTG TAC T 3′). PCR amplified β_2_AR was subcloned into EcoRI and ClaI sites of the C-terminally tagged split-YFP2 BiFC vector [16,28]. Subsequently, β2AR fused to YFP2 was subcloned into EcoRI and XbaI sites of the pEYFP-N1 expression vector containing a kanamycin resistance cassette instead of ampicillin, which is important for efficient selection of Nb clones in *E. coli*. The vectors pBiT2.1-C-β_2_AR-SmBiT and pBiT1.1-N-LgBiT-mini-Gα_i_ were a kind gift from Nevin A. Lambert [30]. To generate pcDNA3-CCR7-SmBiT, SmBiT was first amplified from pAAVS1P-iCLHN (Addgene plasmid #66579, Watertown, MA, USA) [32] by PCR using the following primers: 5′ GCG GTG GAT CGA TTG GAG GTG GCG GTT CTG GTG GTG GCG GTT CCG GCG GTG GCG GTA GCG GCT GGC GGC TGT GCG AAC GCA TTC T 3′ (forward) and 5′ GAA TAG GGC CCT CTA GAT TAG CCC GCC AGA ATG CGT TCG CAC AGC CGC CAG CC 3′ (reverse). CCR7 was amplified from pcDNA3-CCR7-HA [33] using 5′ GAC CCA AGC TTG GTA CCG AGC TCG GAT C 3′ (forward) and 5′ GCC AAT CGA TCC TGG GGA GAA GGT GGT GGT GGT C 3′ (reverse). Both PCR products were cut using ClaI. After ligation of CCR7 to SmBiT, PCR with the respective forward and reverse primer was repeated to insert the resulting PCR product into the pcDNA3 backbone by HindIII and XbaI restriction. The plasmid pcDNA3-CCR7-LgBiT was generated analogously, just that LgBiT was amplified from pAAVS1P-iCLHN using the primers: 5′ GGT GGA TCG ATT GGA GGT GGC GGT TCT GGT GGT GGC GGT TCC GGC GGT GGC GGT AGC ATG GTC TTC ACA CTC G 3′ (forward) and 5′ GAA TAG GGC CCT CTA GAT TAG GTC ACT CCG TTG ATG GTT ACT CGG 3′ (reverse). The expression construct pcDNA3-CCR7-LgBiT was further used to clone various pcDNA3-Nb-LgBiT constructs as well as pcDNA3-Nb80-LgBiT. To this end, both pcDNA3-Nb-YFP1 constructs, including pcDNA3-Nb80-YFP1, and pcDNA3-CCR7-LgBiT were cut using HindIII and ClaI. Afterwards, side directed mutagenesis was performed using primer pairs that were designed with PrimerX (https://www.bioinformatics.org/primerx/). 5′ ACC ATC ACC ATC ACA CGA TTG GAG GTG GCG G 3′ (forward) and 5′ CGC CAC CTC CAA TCG TGT GAT GGT GAT GGT G 3′ (reverse) were used for Nb80-LgBiT whereas 5′ CCA GCC ACC ACA AAA CGA TTG GAG GTG GCG GTT CTG GTG G 3′ (forward) and 5′ CGC CAC CTC CAA TCG TTT TGT GGT GGC TGG ACA CTG TGA C 3′ (reverse) were used for all other Nb-LgBiT constructs. To create Nb-YFP constructs, YFP was PCR amplified from CCR7-YFP using specific primers (5′ AGC AGT AAT CGA TGT GAG CAA GGG CGA GGA 3′ (forward) and 5′ TAG AAT AGG GCC CTC TAG CTA CTT GTA CAG CTC G 3′ (reverse)). YFP1 in pcDNA3-Nb-YFP1 constructs was replaced by complete YFP performing ClaI and ApaI restriction. All primers were custom made by Microsynth (Balgach, Switzerland).

### 4.4. Synthetic Randomization of Nb80 and Construction of a Nb Library into the BiFC Vector

The β_2_AR conformation-specific Nb80 was modified by custom made synthetic randomization (Thermo Fischer Scientific) within CDR1 and CDR3. The synthetically randomized Nb library was amplified by PCR using specific primers (forward: 5′ GAA GGG TAC CAA GCT TGA AAT GGT GCA G 3′; reverse: 5′ GAA GGA GCT CAT CGA TTT TGT GGT GGC 3′). Full length fragments were gel purified und resuspended in TE-buffer revealing a total amount of 11.7 µg of amplified library. The resulting library correctness amounts to 94%. Subsequently, the amplified Nb library was cloned into the C-terminally tagged split-YFP1 BiFC vector using the restriction enzymes HindIII and ClaI.

### 4.5. Fluorescence Associated Cell Sorting (FACS) Based on BiFC

To select Nbs recognizing CCR7, HEK293 cells stably expressing CCR7-YFP2 were transiently transfected with pcDNA3-Nb-library-YFP1. Twenty-four hours after transfection, cells were stimulated with 0.5 µg/mL CCL19 (representing the optimal concentration for inducing cell migration [33,34]) for 20 min at 37°C and 5 % CO_2_. Reconstitution of the two non-fluorescent proteins to native YFP indicating interaction of the Nb with CCR7 was measured by flow cytometry using FACS Aria IIu and the FACSDiva 6 software (BD Biosciences, Franklin Lakes, NJ, USA). BiFC-positive cells were FACS sorted. Beforehand, we conducted a BiFC-negative sorting of HEK293 cells transiently co-expressing pEYFP-N1-β_2_AR-YFP2 and pcDNA3-Nb-library-YFP1, which were stimulated with 10 µM isoproterenol for 20 min at 37°C and 5 % CO_2_, in order to reduce the number of Nbs recognizing β_2_AR. Flow cytometric data were analyzed using the FlowJo10 software (BD Biosciences).

### 4.6. Isolation of Nbs Interacting with CCR7

Plasmids coding for Nbs interacting with CCR7 in the BiFC system were isolated from FACS sorted, transiently transfected HEK293 cells using the DNeasy Blood and Tissue Kit (Qiagen, Hilden, Germany) according to the manufacturer’s protocol (Purification of total DNA from Animal Blood or Cells, Spin-Column Protocol). Isolated plasmid DNA was solved in 200 µl ddH_2_O and subsequently transformed into electro-competent *E. coli* (DH5α) by electroporation. Briefly, approximately 3.5 µg DNA were each added to 500 µl electro-competent *E. coli* and transferred into a pre-cooled electroporation cuvette (0.4 cm gap) (Bio-Rad, Hercules, CA, USA) on ice. After 2 min, electroporation was carried out using the Gene Pulser Xcell Electroporation System (Bio-Rad) applying 3 kV, 25 µF, 200 Ω for ~ 5 ms. Immediately afterwards, 1 mL of SOC medium was added to the bacteria, transferred into a new 2 mL Eppendorf tube and incubated for 1 h at 37 °C while shaking with 450 rpm. Bacteria (200 µl each) were plated on selective agar (LB medium containing 100 µg/mL ampicillin) plates (Ø 14.5 cm) and incubated overnight at 37 °C. The next day, 96 single colonies were picked from one plate and each inoculated in 5 mL selective LB medium. After incubation overnight at 37 °C and constant shaking with 180 rpm, plasmids coding for single Nb clones were isolated from *E. coli* using the NucleoSpin^®^ Plasmid Miniprep Kit (Macherey-Nagel, Düren, Germany), as specified by the manufacturer (protocol for isolation of high copy plasmid DNA from *E. coli*). Additionally, to generate a new library containing Nbs recognizing CCR7, the remaining colonies were harvested from all plates using a cell scratcher and a sufficient amount of medium (8 mL). The resuspended colonies were added to 1 l (total) selective LB medium. Plasmids coding for various Nb clones were isolated from the *E. coli* culture without any further amplification using the NucleoBond^®^ Xtra Midi Kit (Macherey-Nagel) following the manufacturer’s instructions (protocol for high copy plasmid purification (Midi)).

### 4.7. Flow Cytometry Analysis of CCR7-Recognizing Nbs

HEK293 cells were transiently transfected either with CCR7-YFP2 or β_2_AR-YFP2 and various single Nb clones (pcDNA3-Nb-YFP1) and analyzed regarding YFP reconstitution by flow cytometry. Twenty-four hours after transfection, cells were stimulated either with 0.5 µg/mL CCL19 or 10 µM isoproterenol for 20 min. Cells were fixed in 4% formaldehyde (Polysciences, Inc., Warrington, PA, USA) for 15 min at RT. After addition of PBG (3% BSA and 20 mM Glycine in PBS), cells were detached, washed twice with PBS, filtered (70 µm cell strainer) (BD Biosciences) and investigated on a LSRII flow cytometer (BD Biosciences) for BiFC. Flow cytometric data were analyzed using the FlowJo10 software (BD Biosciences).

### 4.8. Split-Luciferase Complementation Assay

The split-luciferase complementation assay was performed to investigate direct interaction between β_2_AR-SmBiT/ CCR7-SmBiT and individual Nb-LgBiT clones. Pilot experiments using different plasmid ratios (2:1, 1:1, 1:2, 1:3) revealed best and most reliable results with minimal background at a plasmid ratio of 1:1 (0.5µg each). Twenty-four hours after transient transfection in a 1:1 plasmid ratio, approximately 6*10^5^ of transfected HEK293 cells were resuspended in 600 µl PBSG (0.05% glucose in PBS) and for each measurement, 80 µl of the cell suspension were transferred onto a 96 well ½ area plate as technical duplicates. After addition of the luminescence substrate coelenterazine H (5 µM), the intensity of luminescence signals was measured on a Spark^®^ Multimode Microplate Reader (Tecan, Männedorf, Switzerland) for 10 min. Subsequently, 10 µM isoproterenol, 1.5 µg/mL CCL19 or PBS was added to the cells and the recording of luminescence signals was continued for 20 min. The highest luminescence signal detected 7 min after stimulation (cycle 34) for the interaction of β_2_AR-SmBiT with Nb80-LgBiT/ CCR7-SmBiT with Nb1-LgBiT was set to 100% and further compared to the luminescence signals of the other Nb-LgBiT clones.

### 4.9. G-Protein Competition Assay Based on Split-Luciferase Complementation

HEK293 cells were transiently transfected with pBiT2.1-C-CCR7-SmBiT, pBiT1.1-N-LgBiT-mini-Gα_i_ as well as individual Nb-YFP1 clones in a plasmid ratio of 1:1:1 and incubated for 48 h. As a proof of concept, HEK293 cells were transiently transfected with pBiT2.1-C-β_2_AR-SmBiT, pBiT1.1-N-LgBiT-mini-Gα_i_ and pcDNA3-Nb80-YFP1. The split-luciferase complementation assay was performed as described in 4.8. Cells were stimulated either with 10 µM isoproterenol or 1.5 µg/mL CCL19. Finally, results were baseline corrected showing the fold increase of luminescence signals after stimulation over the baseline, which is set to 1.

### 4.10. Calcium-Flux

Analysis of chemokine-mediated changes in intracellular free calcium concentrations in H1299-CCR7-HA cells transiently transfected for 24 h with individual Nb-YFP1 clones was performed as previously described [15,33]. Briefly, cells were loaded with fluo-3-AM for 20 min at 37°C, washed and fluorescence changes upon chemokine stimulation were measured on a LSRII flow cytometer (BD Biosciences). Data were analyzed using the FlowJo10 software (BD Biosciences).

### 4.11. CCR7 Endocytosis Assay

HEK293 cells stably expressing CCR7-HA were transiently transfected with various pcDNA3-Nb-YFP clones or pEYFP and pcDNA3-Nb80-YFP serving as negative controls. Twenty-four hours after transfection, cells were left untreated or were stimulated with 1 µg/mL CCL19 (a concentration known to induce maximal receptor internalization [33]) in PBS to induce CCR7 endocytosis and incubated for 30 min at 37°C. Cells were detached and transferred into pre-cooled FACS tubes. Subsequently, remaining CCR7 surface expression was determined by staining cells with anti-human CCR7 APC conjugated antibody for 20 min on ice. Flow cytometry analysis was performed on a LSRII flow cytometer and data were analyzed using FlowJo10 software (BD Biosciences).

### 4.12. Confocal Imaging

For immunofluorescence microscopy, HEK293 cells stably expressing CCR7-YFP2 grown on coverslips were transiently transfected with individual Nb-YFP1 clones. The subsequent day, cells were stimulated or not with 0.5 µg/mL CCL19, washed and fixed in 4% formaldehyde for 15 min at RT. Afterwards, cells were permeabilized using 0.2% Triton-X-100 and 0.125 % SDS in PBG (3% BSA and 20 mM Glycine in PBS) for 30 min at RT and incubated with anti-YFP1 (E385) (abcam, Cambridge, United Kingdom) and anti-YFP2 (11814460001) (Roche, Basel, Switzerland) specific primary antibodies in PBG for 1 h at RT. Cells were washed with PBS and incubated with Alexa Fluor-labeled secondary antibodies (Invitrogen, Life Technologies) for 30 min at RT. Coverslips were mounted using Dako Fluorescence Mounting Medium (Dako, Glostrup, Denmark). Confocal images were acquired on a Leica TCS SP5 II laser scanning microscope using a 63x/1.4 NA oil-immersion objective (Leica, Wetzlar, Germany).

## Abbreviations

BiFC	bimolecular fluorescence complementation
β_2_AR	β_2_-adrenergic receptor
CCR7	CC chemokine receptor 7
CCL19	CC chemokine ligand 19
CCL21	CC chemokine ligand 21
CDR	complementarity determining region
DC	dendritic cell
ERK	extracellular signaling regulated kinase
FACS	fluorescence associated cell sorting
GDP	guanosine diphosphate
GFP	green fluorescent protein
GPCR	G-protein-coupled receptor
GRKs	G-protein-coupled receptor kinases
GTP	guanosine triphosphate
LgBiT	Large BiT
mG-protein	mini-G-protein
Nb	nanobody
Nbs	nanobodies
NLUc	NanoLuc
SEM	standard error of the mean
SmBiT	Small BiT
YFP	yellow fluorescent protein

## References

[1] Griffith J.W., Sokol C.L., Luster A.D. (2014). Chemokines and Chemokine Receptors: Positioning Cells for Host Defense and Immunity. Annu. Rev. Immunol..

[2] Legler D., Thelen M. (2016). Chemokines: Chemistry, Biochemistry and Biological Function. Chim. Int. J. Chem..

[3] Laufer J.M., Legler D.F. (2018). Beyond migration—Chemokines in lymphocyte priming, differentiation, and modulating effector functions. J. Leukoc. Biol..

[4] Marcuzzi E., Angioni R., Molon B., Cal B. (2018). Chemokines and Chemokine Receptors: Orchestrating Tumor Metastasization. Int. J. Mol. Sci..

[5] Kufareva I., Salanga C.L., Handel T.M., Diego S., Jolla L. (2015). Chemokine and chemokine receptor structure and interactions: implications for therapeutic strategies. Immunol. Cell Biol..

[6] Comerford I., Harata-Lee Y., Bunting M.D., Gregor C., Kara E.E., McColl S.R. (2013). A myriad of functions and complex regulation of the CCR7/CCL19/CCL21 chemokine axis in the adaptive immune system. Cytokine Growth Factor Rev..

[7] Förster R., Davalos-Misslitz A.C., Rot A. (2008). CCR7 and its ligands: balancing immunity and tolerance. Nat. Rev. Immunol..

[8] Weber M., Hauschild R., Schwarz J., Moussion C., de Vries I., Legler D.F., Luther S.A., Bollenbach T., Sixt M. (2013). Interstitial dendritic cell guidance by haptotactic chemokine gradients. Science.

[9] Davalos-misslitz A.C.M., Rieckenberg J., Willenzon S., Worbs T., Kremmer E., Bernhardt G., Förster R. (2007). Generalized multi-organ autoimmunity in CCR7- deficient mice. Eur. J. Immunol..

[10] Moschovakis G.L., Förster R. (2012). Multifaceted activities of CCR7 regulate T-cell homeostasis in health and disease. Eur. J. Immunol..

[11] Legler D.F., Uetz-von Allmen E., Hauser M.A. (2014). CCR7: Roles in cancer cell dissemination, migration and metastasis formation. Int. J. Biochem. Cell Biol..

[12] Legler D.F., Thelen M. (2018). New insights in chemokine signaling. F1000Research.

[13] Legler D.F., Matti C., Laufer J.M., Jakobs B.D., Purvanov V., Allmen E.U., Thelen M. (2017). Modulation of Chemokine Receptor Function by Cholesterol: New Prospects for Pharmacological Intervention. Mol. Pharmacol..

[14] Hauser M.A., Legler D.F. (2016). Common and biased signaling pathways of the chemokine receptor CCR7 elicited by its ligands CCL19 and CCL21 in leukocytes. J. Leukoc. Biol..

[15] Purvanov V., Matti C., Samson G.P.B., Kindinger I., Legler D.F. (2018). Fluorescently Tagged CCL19 and CCL21 to Monitor CCR7 and ACKR4 Functions. Int. J. Mol. Sci..

[16] Hauser M.A., Schaeuble K., Kindinger I., Impellizzieri D., Krueger W.A., Hauck C.R., Boyman O., Legler D.F. (2016). Inflammation-Induced CCR7 Oligomers Form Scaffolds to Integrate Distinct Signaling Pathways for Efficient Cell Migration. Immunity.

[17] Laufer J.M., Kindinger I., Artinger M., Pauli A., Legler D.F. (2018). CCR7 Is Recruited to the Immunological Synapse, Acts as Co-stimulatory Molecule and Drives LFA-1 Clustering for Efficient T Cell Adhesion Through ZAP70. Front. Immunol..

[18] Tesmer J.J.G. (2016). Hitchhiking on the heptahelical highway: structure and function of 7 TM receptor complexes. Nat. Rev. Mol. Cell Biol..

[19] Zhao C., Fenalti G., Wu H., Han G.W., Cherezov V. (2015). Crystal structure of the chemokine receptor CXCR4 in complex with a viral chemokine. Sience.

[20] Zheng Y., Han G.W., Abagyan R., Cherezov V., Kufareva I., Handel T.M., Zheng Y., Han G.W., Abagyan R., Wu B. (2017). Structure of CC Chemokine Receptor 5 with a Potent Chemokine Antagonist Reveals Mechanisms of Chemokine Recognition and Molecular Mimicry by HIV Article Structure of CC Chemokine Receptor 5 with a Potent Chemokine Antagonist Reveals Mechanisms of Chemokin. Immunity.

[21] Rasmussen S.G.F., Choi H., Fung J.J., Pardon E., Casarosa P., Chae P.S., Devree B.T., Rosenbaum D.M., Thian F.S., Kobilka T.S. (2011). Structure of a nanobody-stabilized active state of the b 2 adrenoceptor. Nature.

[22] Irannejad R., Tomshine J.C., Tomshine J.R., Chevalier M., Mahoney J.P., Steyaert J., Rasmussen S.G.F., Sunahara R.K., El-Samad H., Huang B. (2013). Conformational biosensors reveal GPCR signalling from endosomes. Nature.

[23] Manglik A., Kobilka B.K., Steyaert J. (2017). Nanobodies to Study G Protein – Coupled Receptor Structure and Function. Annu. Rev. Pharmacol. Toxicol..

[24] Heukers R., De Groof T.W.M., Smit M.J. (2019). ScienceDirect Nanobodies detecting and modulating GPCRs outside in and inside out. Curr. Opin. Cell Biol..

[25] Revets H., De Baetselier P., Muyldermans S. (2005). Nanobodies as novel agents for cancer therapy. Expert Opin. Biol. Ther..

[26] Steyaert J., Kobilka B.K. (2011). Nanobody stabilization of G protein-coupled receptor conformational states. Curr. Opin. Struct. Biol..

[27] De Wit R.H., Verkaar F., Smit M.J., Mujic A. (2014). GPCR-targeting nanobodies: attractive research tools, diagnostics, and therapeutics. CellPress.

[28] Nyfeler B., Michnick S.W., Hauri H.-P. (2005). Capturing protein interactions in the secretory pathway of living cells. Proc. Natl. Acad. Sci..

[29] Staus D.P., Wingler L.M., Strachan R.T., Rasmussen S.G.F., Pardon E., Ahn S., Steyaert J., Kobilka B.K., Lefkowitz R.J. (2014). Regulation of β2-adrenergic receptor function by conformationally selective single-domain intrabodies. Mol. Pharmacol..

[30] Wan Q., Okashah N., Inoue A., Nehme R., Carpenter B., Tate C.G., Lambert N.A. (2018). Mini G protein probes for active G protein– coupled receptors (GPCRs) in live cells. J. Biol. Chem..

[31] Schaeuble K., Hauser M.A., Rippl A.V., Bruderer R., Otero C., Groettrup M., Legler D.F. (2012). Ubiquitylation of the chemokine receptor CCR7 enables efficient receptor recycling and cell migration. J. Cell Sci..

[32] Cerbini T., Funahashi R., Luo Y., Liu C., Park K., Rao M., Malik N., Zou J. (2015). Transcription activator-like effector nuclease (TALEN)-mediated CLYBL targeting enables enhanced transgene expression and one-step generation of dual reporter human induced pluripotent stem cell (iPSC) and neural stem cell (NSC) lines. PLoS ONE.

[33] Otero C., Groettrup M., Legler D.F. (2006). Opposite fate of endocytosed CCR7 and its ligands: recycling versus degradation. J. Immunol..

[34] Otero C., Eisele P.S., Schaeuble K., Groettrup M., Legler D.F. (2008). Distinct motifs in the chemokine receptor CCR7 regulate signal transduction, receptor trafficking and chemotaxis. J. Cell Sci..

